# Enhanced Magnetic Anisotropies of Single Transition-Metal Adatoms on a Defective MoS_2_ Monolayer

**DOI:** 10.1038/srep09361

**Published:** 2015-03-23

**Authors:** W. T. Cong, Z. Tang, X. G. Zhao, J. H. Chu

**Affiliations:** 1 Key Laboratory of Polar Materials and Devices, Ministry of Education of China, East China Normal University, Shanghai 200241, People's Republic of China

## Abstract

Single magnetic atoms absorbed on an atomically thin layer represent the ultimate limit of bit miniaturization for data storage. To approach the limit, a critical step is to find an appropriate material system with high chemical stability and large magnetic anisotropic energy. Here, on the basis of first-principles calculations and the spin-orbit coupling theory, it is elucidated that the transition-metal Mn and Fe atoms absorbed on disulfur vacancies of MoS_2_ monolayers are very promising candidates. It is analysed that these absorption systems are of not only high chemical stabilities but also much enhanced magnetic anisotropies and particularly the easy magnetization axis is changed from the in-plane one for Mn to the out-of-plane one for Fe by a symmetry-lowering Jahn-Teller distortion. The results point out a promising direction to achieve the ultimate goal of single adatomic magnets with utilizing the defective atomically thin layers.

Single magnetic atoms absorbed on a surface represent the ultimate limit of bit miniaturization for the magnetic data storage^1^,^2^. To achieve this ideal goal, a critical step is to find an appropriate material system in which there are not only strong enough binding between the absorption atoms and the substrate surface to guarantee the system's chemical stability but also large enough magnetic anisotropic energy (MAE) to ensure the magnetic stability of the atomic magnets over thermal fluctuations and spin decoherence. After intensive studies in the last decade^3^,^4^,^5^, impressive progresses have been achieved towards the goal. It has been elucidated both experimentally and theoretically that, because of symmetry breaking and hybridization, the MAE of the transition metal adatoms, such as Co on a Pt surface^3^, Fe or Mn on a CuN surface^4^, etc., can be greatly enhanced.

Recently, discovery of graphene^6^ has raised exciting prospects to approaching the ultimate goal by utilizing the atomically thin layers as the substrate^7^. It has been predicted that single magnetic atoms absorbed on graphene as well as hexagonal boron nitride (h-BN) are of huge uniaxial magnetic anisotropies^8^,^9^,^10^,^11^,^12^ and experimentally, a large magnetic anisotropy of 8.1 meV or about 3.0 meV has been observed for Co adatoms on graphene^13^ or on Cu_2_N surface^14^. However, as a consequence of the single atom thickness of both graphene and h-BN and their strong covalent binding in plane, the implanted transition metal atoms significantly stick out of the plane of the host material, which leads to a chemical instability if the system is exposed to the air.

Monolayer molybdenum disulfide (MoS_2_) is another atomic-scale thin layer material. It is a prototypical quasi-two-dimensional crystal consisting of two close-packed S layers separated by a Mo atomic layer. Different from graphene, the MoS_2_ monolayer is a direct band-gap semiconductor^15^. Recently, high quality MoS_2_ monolayers with large areas have been achieved by using the chemical vapor deposition technology^16^,^17^,^18^, which paves the way to a number of promising applications of the MoS_2_ monolayer in semiconductor optoelectronic and spintronic devices. For instance, a transistor with a high mobility at room temperature^19^ and nonvolatile memory cells^20^ based on monolayer MoS_2_ have been successfully fabricated. Moreover, experiments have shown that various structural defects occur in the MoS_2_ monolayers^21^,^22^, which provide further exciting opportunities to tailor the local properties of the substrate to create new functionalities.

For this reason, we investigated the stabilities and the MAE's of the single transition-metal atoms (Mn and Fe) absorbed on the pristine and defective MoS_2_ monolayers by first-principles calculations. By combining the spin-orbit coupling theory, it is elucidated that the TM atoms absorbed on the disulfur vacancies of the MoS_2_ monolayer are of high chemical stabilities as well as much enhanced magnetic anisotropic energies. Particularly, it is analysed that, as a consequence of a symmetry-lowering Jahn-Teller distortion, the easy axis of magnetization can be changed from the one on the MoS_2_ plane to the one out of the plane by changing the absorption adatom from Mn to Fe. These results indicate that the single TM adatoms absorbed on the defects of the MoS_2_ monolayers are very promising candidates for fabricating the atomic-scale magnetic bit for data storage.

## Results

**Figure 1:**
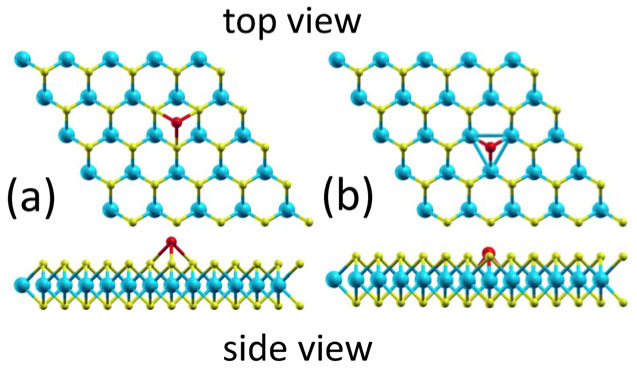
Top and side views of the optimized geometries of the Fe adatom absorbed on the pristine MoS_2_ monolayer (a) and on its disulfur vacancies (b). The cyan, yellow, and red balls denote Mo, S, and Fe atoms, respectively.

**Figure 2:**
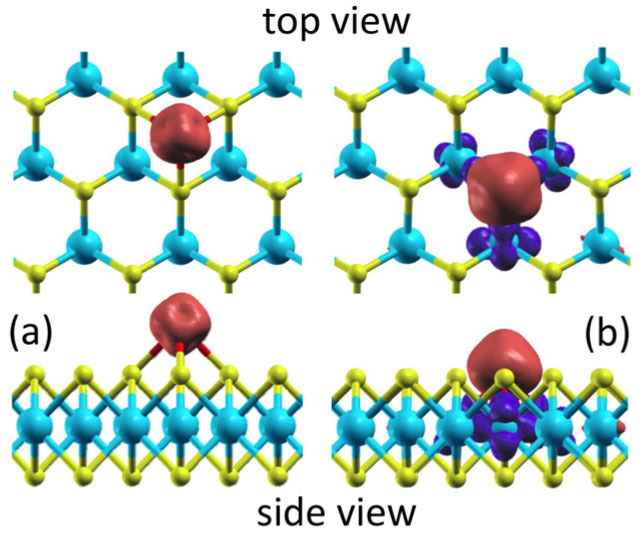
Enlarged isosurfaces of the calculated spin density distributions for the Fe adatom absorbed on the pristine MoS_2_ monolayer (a) and on its disulfur vacancies (b) (upper panel: top view, lower panel: side view).

At first, we calculated the stability and the MAE of a single Fe or Mn absorbed on a pristine MoS_2_ monolayer. There are several possible absorption positions for the TM adatom on the MoS_2_ monolayer. We found that, similar to a TM atom absorbed on graphene^23^, the most stable absorption site is also the octahedron site as shown in Figure 1a. At this site, the binding energy of a single Fe (Mn) adatom is 1.34 (1.18) eV and the calculated total magnetic moment of the absorption system is 4.0 (5.0) *μ*_B_. As demonstrated by the spin density distributions shown in Figure 2a, the magnetic moments are mainly contributed from the TM adatoms. The obtained high spin configurations agree with the Hund's rule.

However, as presented in Table 1, the MAE's of the TM adatoms on the pristine MoS_2_ monolayer are rather lower, only −0.2 and −0.3 meV for the Mn and Fe, respectively (the negative sign indicating an out-of-plane magnetic anisotropic axis). Particularly, the optimized structure shown in Figure 1 indicates that, although the binding energy of an absorbed Fe atom on the MoS_2_ monolayer is 1.34 eV, the adatom is still lying far above the layer (about 1.75 Å from the top S layer), indicating that the system may be unstable when exposed to air. Similar situations are obtained for the Mn adatom as well (Table 1). These results imply that the TM adatoms absorbed on the pristine MoS_2_ monolayers are not the ideal material systems.

**Figure 3:**
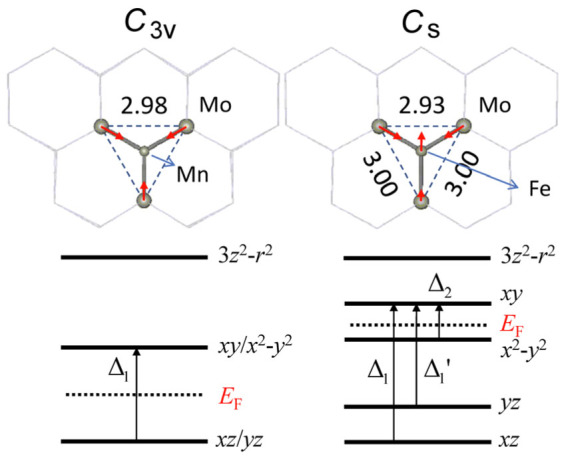
Enlarged optimized geometries around the Mn (left panel) and Fe (right panel) adatoms absorbed on the disulfur vacancies of the defective MoS_2_ monolayer, together with sketches of the localized impurity levels around *E*_F_ and their dominant 3*d* atomic orbital characteristics.

We then studied the stabilities and the MAE's of the TM adatoms absorbed on the defective MoS_2_ monolayers. Zhou et al.^22^ have shown that the as-grown monolayer MoS_2_ is very defective and by using an aberration-corrected scanning transmission electron microscope a number of intrinsic defects are identified, such as a disulfur vacancy (V_S2_) with a pair of S atoms (i.e., a S_2_ column) missing and an antisite Mo to the V_S2_ (Mo_S2_), etc. It is thereby natural to consider replacing the antisite Mo atom in the Mo_S2_ by a different TM atom, which is effectively equivalent to the case that the TM atom is absorbed on the V_S2_ site. Figure 1b presents the optimized structure of a Fe atom absorbed on the V_S2_ site and details about the local atomic structures around the Mn and Fe adatoms are presented in the enlarged geometries shown in Figure 3. It is found that the absorption of the TM atoms on the V_S2_ causes significant rearrangements of the atoms around the defects. For the case of the Mn adatom on the V_S2_, three Mo atoms in the first-nearest neighboring sites to the defect symmetrically relax inward to the defect center by 0.10 Å and the absorption system still possesses a *C*_3v_ symmetry [Figure 3a]. In the case of the Fe absorbed on the V_S2_, a symmetry-lowering distortion is observed with two Mo relaxed inward by 0.13 Å and another one relaxed inward by 0.07 Å so that the system's symmetry reduces to *C*_s_.

The calculated binding energies of the Mn and Fe adatoms are 3.50 and 3.51 eV, respectively, while the calculated total magnetic moments of the Mn and Fe absorption systems are, respectively, 3.0 and 2.0 *μ*_B_ (Table 1). Similarly, the magnetic moments are mainly contributed from the TM adatoms as well (Figure 2). For instance, the net magnetic moment of the Fe adatom is 3.0 *μ*_B_ (Table 1). The decomposed Mulliken charges indicate that the Fe adatom is of 4.8 (1.8) 3*d* electrons with the majority (minority) spin. Because the total number of 3*d* electrons (6.6) in the Fe adatom exceeds that of an isolated Fe atom (6.0), there must be orbital-hybridization-induced 3*d* electron transform from the surrounding Mo atoms, which results in a net magnetic moment of −1.0 *μ*_B_ in the surrounding Mo atoms (Figure 2). Moreover, as a consequence of the stronger 3*d* orbital hybridization (Figure 2), the binding energy of a Fe (Mn) adatom on the V_S2_ increases to 3.51 (3.50) eV, more than doubled compared to that of a Fe adatom (1.34 eV) or Mn adatom (1.18 eV) on the pristine MoS_2_ monolayer. The optimized structure presented in Figure 1b shows that the TM atoms absorbed on the V_S2_ defects only slightly stick out of the top sulfur layer by 0.28 Å (Mn) or 0.34 Å (Fe), implying that the TM adatoms are stable in various application environments, such as exposed to air.

The most interesting property revealed by the present calculations is the enhanced MAE's of the TM adatoms on the V_S2_ defects. For the Mn adatom, a large MAE of 1.3 meV with the easy axis of magnetization on the MoS_2_ plane is obtained, while for the Fe adatom an even larger MAE of −3.6 meV with the out-of-plane easy axis is observed. So far, the highest MAE record of the TM adatoms is −9.3 meV and such a giant perpendicular MAE was observed on the single Co atoms absorbed on Pt (111) surface by Gambardella et al.^3^. Similarly, the large values of MAE were observed for the Co adatoms on Cu_2_N surface by Oberg et al.^14^. We also calculated the MAE of a Co adatom absorbed on the pristine MoS_2_ monolayer and on its V_S2_ defect and the perpendicular MAE of 0.7 and 4.2 meV were obtained, respectively. Although the enhanced MAE of −3.6 meV (Fe) or −4.2 meV (Co) obtained in the present work is still less than half of the current record, it points out a promising direction with utilizing the defective atomically thin layers. Particularly, from the experimental point of view, a large out-of-plane MAE is more helpful to achieve the single atom bit on surfaces. The present calculations indicate that both Fe and Co adatoms absorbed on the defective MoS_2_ monolayer are of the out-of-plane MAE and thus they are the promising candidates for single-atom data storage.

## Discussion

Next, we analysed the calculated MAE's and discuss their physical origin based on the molecular orbital (MO) theory and the spin-orbit coupling (SOC) interaction. According to the theory of Bruno^24^, the MAE is determined by the anisotropic orbital moments restored by the spin-orbit coupling interaction, 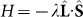 (here *λ* is the SOC constant). For the TM adatoms, as the electrostatic interaction of the surrounding ions is larger than the SOC interaction, the latter is treated as a perturbation. Thereby, the energy gain induced by the SOC to the first order reads^25^,^26^, 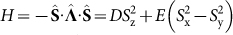. Here, the spin-flip terms are neglected^27^, 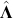 is the tensor of the SOC, *D* = *Λ*_zz_ − (*Λ*_xx_ + *Λ*_yy_)/2 and *E* = (*Λ*_xx_ − *Λ*_yy_)/2. *Λ*_xx_, *Λ*_yy_ and *Λ*_zz_ are the diagonal elements of the SOC tensor given by
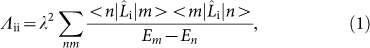
where 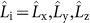 stands for three anisotropic components of the orbital moment operator 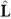, |*n*> and *E_n_* (|*m*> and *E_m_*) are the wavefunctions and the corresponding eigen-energies of the occupied (unoccupied) electron states, respectively, and if omitting the prefactor *λ*^2^, the diagonal elements *Λ*_ii_ are actually the expected values of the anisotropic orbital moment operator 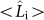 to the first order of perturbation, i.e., the unquenched orbital moment^24^,^28^.

The above Eq. (1) indicates that the localized electronic states around the Fermi energy level are of dominant effects to the calculated unquenched orbital moments. These localized impurity/adatom states are formed by the hybridizations of the 3*d* orbitals of the TM adatoms with those of the surrounding Mo atoms. Because the net magnetic moments of the absorption systems are mainly contributed from the TM adatoms, we here consider only the SOC of the TM adatoms and thereby their unquenched orbital moments can be rewritten as
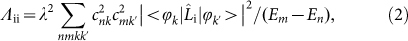
where *c_nk_* (*c_mk_*_′_) is the LCPAO coefficient of the primary 3*d* pseudo atomic orbital (PAO) *ϕ_k_* (*ϕ_k_*_′_) of the TM adatom in the state |*n*> (|*m*>) obtained from the first-principles calculations.

**Figure 4:**
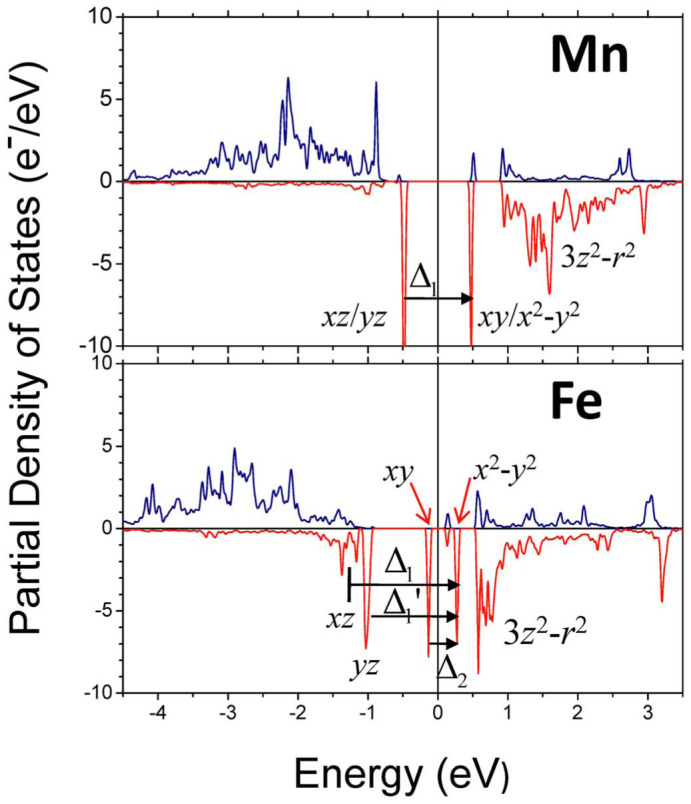
Partial density of states (PDOS) projected to the Mn (upper panel) and Fe (lower panel) adatoms absorbed on the disulfur vacancies of the defective MoS_2_ monolayer. Positive (negative) PDOS denotes the majority (minority) spin state.

Figure 4 presents the calculated partial density of states (PDOS) projected to the Mn and Fe adatoms on the V_S2_ site. As shown in Figure 4, for the case of the Mn adatom, two localized energy levels with the minority spin around the *E*_F_ have to be taken into account in the calculations of *Λ*_ii_ [as the majority-spin states are almost fully occupied (Figure 4), their contribution to *Λ*_ii_ is negligible]. Because the Mn adatom on the V_S2_ possesses the *C*_3v_ symmetry, the localized states are doubly degenerate and the one below (above) *E*_F_ is dominated by the Mn adatom's *d_xz_* (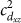 ~ 0.45) and *d_yz_* (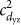 ~ 0.45) orbitals [*d_xy_* (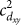 ~ 0.41) and 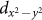 (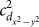 ~ 0.41) orbitals]. Therefore, only the following items have nonzero contributions to *Λ*_ii_, i.e., 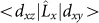, 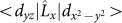, 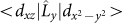, and 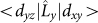, so that *Λ_zz_* = 0, 
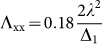
, and 
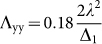
. Based on the calculated *Λ*_ii_, we get that 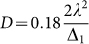, *E* = 0, and the MAE of the Mn adatom on the V_S2_ as 
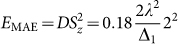
. The SOC constants *λ* for the TM elements are usually around 10–50 meV^26^ and here by taking *λ* = 30 meV as a parameter, Δ_1_ = 0.96 eV (Figure 4), the MAE is estimated to be 1.35 meV with the easy axis on the MoS_2_ monolayer. This result is consistent with 1.3 meV of the first-principles calculation (Table 1).

It has to be aware of that, as shown by the theoretical analyses for the magnetic properties of single holmium atoms on Pt(111) surface^29^, the in-plane SOC terms in the case of the C_3v_ symmetry have only vanishing contributions to the MAE. Our perturbation calculation shows that the in-plane SOC term 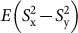 is effectively zero due to the exact cancelling effect between the diagonal elements *Λ_xx_* and *Λ_yy_* of the SOC tensor under the C_3v_ symmetry. To verify these symmetric analyses for the MAE, we also calculated the MAE's of the Mn adatom on the V_S2_ defect with two different in-plane magnetization directions, i.e., along the *x* axis and the *y* axis, respectively. In both the cases the same MAE of 1.3 meV are obtained, which are consistent with the symmetric analyses.

When the TM adatom is changed from Mn (3*d*^5^) to Fe (3*d*^6^), the number of the *d* electrons increases by one and the upper empty energy level above the *E*_F_ is now half occupied. As a result, the symmetry-lowering Jahn-Teller distortion is expected. As shown in Figure 3, the symmetry of the TM adatom is reduced from *C*_3v_ for the Mn adatom to *C*_s_ for the Fe adatom and the degeneracy of both the upper and lower levels is lifted as well (Figures 3 and 4). With use of the four localized energy levels split from the upper and lower levels as shown in Figures 3 and 4 and their 3*d* PAO's LCPAO coefficients (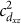 ~ 0.60, 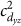 ~ 0.68, 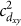 ~ 0.46, and 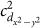 ~ 0.41), we get 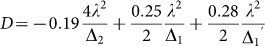 and 
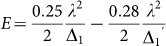
 with the transition energies of Δ_1_ = 1.51 eV, Δ_1_′ = 1.27 eV, and Δ_2_ = 0.39 eV, respectively. Thereby, the MAE of the Fe adatom on the V_S2_ is estimated as 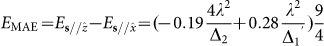, i.e., *E*_MAE_ = −3.5 meV (here *λ* = 30 meV is also employed, implying that we omit the difference in the SOC constants for these two TM adatoms). The result indicates that the easy axis of magnetization is perpendicular to the MoS_2_ plane and the obtained MAE value agrees well with the first-principles calculation. It is noteworthy that, as 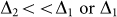, both the sign and the value of the MAE is dominated by the transition from *d_xy_* to 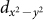 with the energy of Δ_2_ (Figure 3). As a matter of fact, the first item associated with Δ_2_ in *E*_MAE_ contributes −4.0 meV to the estimated MAE (−3.5 meV), elucidating that the energy level splitting induced by the Jahn-Teller distortion plays a very essential role in enhancing the magnetic anisotropy and in tuning its easy axis.

In conclusion, the stabilities and the magnetic anisotropic energies of the single transition-metal (TM) atoms (Mn and Fe) absorbed on the pristine and defective MoS_2_ monolayers are studied on the basis of the first-principles calculations and the spin-orbit coupling theory. It is elucidated that the TM atoms absorbed on the disulfur vacancies of the MoS_2_ monolayer are of high chemical stabilities as well as much enhanced magnetic anisotropic energies. Particularly, it is analysed that the easy magnetization axis of the atomic magnets is changed by the symmetry-lowering Jahn-Teller distortion. These results indicate that the single TM adatoms absorbed on the defects of the MoS_2_ monolayers are very promising candidates for fabricating the atomic-scale magnetic bit for data storage.

## Methods

To simulate the transition metal adatoms on the pristine and defective MoS_2_ monolayers, an 75-site slab supercell of MoS_2_ with two vacuum layers of 20 Å on both the top and down sides of the MoS_2_ layer were constructed. The first-principles calculations were performed based on the spin-polarized density functional theory within the generalized gradient approximation (GGA). The electron-ion interactions are presented by the Troullier-Martins-type norm-conserving pseudopotentials^30^ with a partial core correction and the GGA exchange-correlation potential in the form of Perdew-Burke-Ernzerhof (PBE) functional is adopted. The electron wavefunctions are expanded as linear combinations of pseudo atomic orbitals (LCPAOs)^31^, which are generated by using a confinement potential scheme^32^,^33^ with the cutoff radius of 7.0, 7.0, 6.0, and 6.0 a.u. for Mo, S, Fe, and Mn, respectively. In the self-consistent calculations of charge density a 9 × 9 × 1 Monkhorst-Pack *k* grid is employed and in the structure relaxations the atomic geometries are fully optimized until the Hellmann-Feynman forces are less than 0.01 eV/Å.

The stability of the adatoms on the MoS_2_ monolayers is characterized by their binding energy given by *E*_B_ = *E*[MoS_2_] + *μ*_TM_ − *E*[TM + MoS_2_]. For the TM adatom on the pristine MoS_2_, *E*[TM + MoS_2_] and *E*[MoS_2_] are, respectively, the calculated system total energies of the pristine MoS_2_ absorbed with the TM adatom and of the pristine MoS_2_ only. While for the TM adatom on the defective MoS_2_, *E*[TM + MoS_2_] and *E*[MoS_2_] are the calculated system total energies of the defective MoS_2_ absorbed with the TM adatom and of the defective MoS_2_, respectively. *μ*_TM_ is the chemical potential of the transition metal adatom, which is obtained as the calculated total energy of the single isolated TM atom in the same supercell (in the absence of MoS_2_). The MAE is calculated as *E*_MAE_ = *E*[*θ* = 0°] − *E*[*θ* = 90°]^25^, where *θ* denotes the angle of the spin polarization with respect to the *c* axis (namely the perpendicular direction of the MoS_2_ monolayer) and the total energies are obtained by means of the self-consistent full-relativistic calculations^34^ with a converging standard of 1.0 × 10^−6^ eV. To verify the plausible anisotropy of different in-plane directions, the in-plane total energies are further calculated with the magnetization direction fixed to the *x*-axis (*θ* = 90°, *φ* = 0°) and the *y*-axis (*θ* = *φ* = 90°), respectively.

## Author Contributions

Z.T. conceived the idea, designed the research, and prepared the manuscript. W.T.C. performed the calculations. W.T.C., X.G.Z. and J.H.C. contributed to the analysis/interpretation of the results and the preparation/revision of the manuscript.

## Figures and Tables

**Table 1:** Calculated binding energies (*E*_B_), distances to the top sulfur layer (Δh), total magnetic moments (m.m.), and magnetic anisotropic energies (MAE) of the Mn and Fe adatoms absorbed on the pristine MoS_2_ monolayer and its disulfur vacancies (V_S2_). The values in the parentheses are the magnetic moments of the adatoms

Absorption systems	*E*_B_ (eV)	Δh (Å)	m.m. (*μ*_B_)	MAE (meV)
Fe on pristine MoS_2_	1.34	1.75	4.0 (4.0)	−0.3
Mn on pristine MoS_2_	1.18	1.77	5.0 (5.0)	−0.2
Fe on V_S2_ of MoS_2_	3.51	0.28	2.0 (3.0)	−3.6
Mn on V_S2_ of MoS_2_	3.50	0.34	3.0 (4.0)	1.3

## References

[b1] Gambardella P., Rusponi S., Cren T., Weiss N. & Brune H. Magnetic Anisotropy from Single Atoms to Large Monodomain Islands of Co/Pt(111). C. R. Physique 6, 75–87 (2005).

[b2] Carbone C. *et al.* Self-Assembled Nanometer-Scale Magnetic Networks on Surfaces: Fundamental Interactions and Functional Properties. Adv. Funct. Mater. 21, 1212–1228 (2011).

[b3] Gambardella P. *et al.* Giant Magnetic Anisotropy of Single Cobalt Atoms and Nanoparticles. Science 300, 1130 (2003). 12750516 10.1126/science.1082857

[b4] Hirjibehedin C. F. *et al.* Large Magnetic Anisotropy of a Single Atomic Spin Embedded in a Surface Molecular Network. Science 317, 1199 (2003). 17761877 10.1126/science.1146110

[b5] Bryant B., Spinelli A., Wagenaar J. J. T., Gerrits M. & Otte A. F. Local Control of Single Atom Magnetocrystalline Anisotropy. Phys. Rev. Lett. 111, 127203 (2013). 24093296 10.1103/PhysRevLett.111.127203

[b6] Novoselov K. S. *et al.* Electric Field Effect in Atomically Thin Carbon Films. Science 306, 666 (2004). 15499015 10.1126/science.1102896

[b7] Xie L. M., Ling X., Fang Y., Zhang J. & Liu Z. F. Graphene as a Substrate to Suppress Fluorescence in Resonance Raman Spectroscopy. J. Am. Chem. Soc. 131, 9890–9891 (2009). 19572745 10.1021/ja9037593

[b8] Xiao R. J. *et al.* Co Dimers on Hexagonal Carbon Rings Proposed as Subnanometer Magnetic Storage Bits. Phys. Rev. Lett. 103, 187201 (2009). 19905826 10.1103/PhysRevLett.103.187201

[b9] Zhou Y. G., Zu X. T., Gao F., Lv H. F. & Xiao H. Y. Adsorption-Induced Magnetic Properties and Metallic Behavior of Graphene. Appl. Phys. Lett. 95, 123119 (2009).

[b10] Xiao R. J., Kuz'min M. D., Koepernik K. & Richter M. CoIr-Carbon Complexes with Magnetic Anisotropies Larger than 0.2 eV: A Density-Functional-Theory Prediction. Appl. Phys. Lett. 97, 232501 (2010).

[b11] Kandpal H. C., Koepernik K. & Richter M. Strong Magnetic Anisotropy of Chemically Bound Co Dimers in a Graphene Sheet. Phys. Rev. B 86, 235430 (2012).

[b12] Lu Y., Zuo X., Feng M. & Zhou T. Magnetic Anisotropy in the Boron Nitride Monolayer Doped by 3d Transitional Metal Substitutes at Boron-Site. J. Appl. Phys. 113, 17C304 (2013).

[b13] Donati F. *et al.* Magnetic Moment and Anisotropy of Individual Co Atoms on Graphene. Phys. Rev. Lett. 111, 236801 (2013). 24476294 10.1103/PhysRevLett.111.236801

[b14] Oberg J. C. *et al.* Control of single-spin magnetic anisotropy by exchange coupling. Nature Nanotechnol. 9, 64–68 (2014). 24317285 10.1038/nnano.2013.264

[b15] Mak K. F., Lee C., Hone J., Shan J. & Heinz T. F. Atomically Thin MoS_2_: A New Direct-Gap Semiconductor. Phys. Rev. Lett. 105, 136805 (2010). 21230799 10.1103/PhysRevLett.105.136805

[b16] Lee Y. H. *et al.* Synthesis of Large-Area MoS_2_ Atomic Layers with Chemical Vapor Deposition. Adv. Mater. 24, 2320–2325 (2012). 22467187 10.1002/adma.201104798

[b17] Zhan Y., Liu Z., Najmaei S., Ajayan P. M. & Lou J. Large Area Vapor Phase Growth and Characterization of MoS_2_ Atomic Layers on SiO_2_ Substrate. Small 8, 966–971 (2012). 22334392 10.1002/smll.201102654

[b18] Liu K. K. *et al.* Growth of Large-Area and Highly Crystalline MoS_2_ Thin Layers on Insulating Substrates. Nano Lett. 12, 1538–1544 (2012). 22369470 10.1021/nl2043612

[b19] Radisavljevic B., Radenovic A., Brivio J., Giacometti V. & Kis A. Single-Layer MoS_2_ Transistors. Nat. Nanotechnol. 6, 147–150 (2011). 21278752 10.1038/nnano.2010.279

[b20] Bertolazzi S., Krasnozhon D. & Kis A. Nonvolatile Memory Cells Based on MoS_2_/Graphene Heterostructures. ACS Nano 7, 3246–3252 (2013). 23510133 10.1021/nn3059136

[b21] Komsa H. P. *et al.* Two-Dimensional Transition Metal Dichalcogenides under Electron Irradiation: Defect Production and Doping. Phys. Rev. Lett. 109, 035503 (2012). 22861869 10.1103/PhysRevLett.109.035503

[b22] Zhou W. *et al.* Intrinsic Structural Defects in Monolayer Molybdenum Disulfide. Nano Lett. 13, 2615–2622 (2013). 23659662 10.1021/nl4007479

[b23] Cha K. T., Neaton J. B. & Cohen M. L. First-Principles Study of Metal Adatom Adsorption on Graphene. Phys. Rev. B 77, 235430 (2008).

[b24] Bruno P. Tight-Binding Approach to the Orbital Magnetic Moment and Magnetocrystalline Anisotropy of Transition-Metal Monolayers. Phys. Rev. B. 39, 865 (1989). 10.1103/physrevb.39.865 9947253

[b25] Stöhr J. & Siegmann H. C. in Magnetism: From Fundamentals to Nanoscale Dynamics, (Springer-Verlag, Berlin, 2006).

[b26] Loubser J. H. N. & van Wyk J. A. Electron Spin Resonance in the Study of Diamond. Rep. Prog. Phys. 41, 1201 (1978).

[b27] van der Laan G. Microscopic origin of magnetocrystalline anisotropy in transition metal thin films. J. Phys.: Condens. Matter 10, 3239–3253 (1998).

[b28] Stöhr J. Exploring the Microscopic Origin of Magnetic Anisotropies with X-ray Magnetic Circular Dichroism (XMCD) Spectroscopy. J. Magn. Magn. Mater. 200, 470 (1999).

[b29] Miyamachi T. *et al.* Stabilizing the magnetic moment of single holmium atoms by symmetry. Nature 503, 242–246 (2013). 24226888 10.1038/nature12759

[b30] Troullier N. & Martins J. L. Efficient Pseudopotentials for Plane-Wave Calculations. Phys. Rev. B 43, 1993–2006 (1991). 10.1103/physrevb.43.1993 9997467

[b31] Ozaki T. Variationally Optimized Atomic Orbitals for Large-Scale Electronic Structures. Phys. Rev. B 67, 155108 (2003).

[b32] Ozaki T. & Kino H. Variationally Optimized Basis Orbitals for Biological Molecules. J. Chem. Phys. 121, 10879 (2004). 15634039 10.1063/1.1794591

[b33] Ozaki T. & Kino H. Numerical Atomic Basis Orbitals from H to Kr. Phys. Rev. B 69, 195113 (2004).

[b34] Theurich G. & Hill N. A. Self-Consistent Treatment of Spin-Orbit Coupling in Solids using Relativistic Fully Separable *ab initio* Pseudopotentials. Phys. Rev. B 64, 073106 (2001).

